# The epidemiology of multidrug-resistant organisms in persons diagnosed with cancer in Norway, 2008–2018: expanding surveillance using existing laboratory and register data

**DOI:** 10.1007/s10096-023-04698-3

**Published:** 2023-11-18

**Authors:** Anders Skyrud Danielsen, Petter Elstrøm, Hanne-Merete Eriksen-Volle, Solveig Hofvind, David W. Eyre, Oliver Kacelnik, Jørgen Vildershøj Bjørnholt

**Affiliations:** 1 Department of Microbiology, Oslo University Hospital, Oslo, Norway; 2 Institute of Clinical Medicine, University of Oslo, Oslo, Norway; 3 Centre for Epidemic Intervention Research, Norwegian Institute of Public Health, Oslo, Norway; 4 Department of Infection Control and Preparedness, Norwegian Institute of Public Health, Oslo, Norway; 5 Cancer Registry of Norway, Oslo, Norway; 6 Big Data Institute, University of Oxford, Oxford, UK

**Keywords:** MDRO, MRSA, VRE, 3GCR-E, Cancer, Surveillance

## Abstract

Surveillance has revealed an increase of multidrug-resistant organisms (MDROs), even in low-prevalent settings such as Norway. MDROs pose a particular threat to at-risk populations, including persons with cancer. It is necessary to include such populations in future infection surveillance. By combining existing data sources, we aimed to describe the epidemiology of MDROs in persons diagnosed with cancer in Norway from 2008 to 2018. A cohort was established using data from the Cancer Registry of Norway, which was then linked to notifications of methicillin-resistant *Staphylococcus aureus* (MRSA), vancomycin- and/or linezolid-resistant enterococci (V/LRE), and carbapenemase-producing Gram-negative bacilli (CP-GNB) from the Norwegian Surveillance System for Communicable Diseases, and laboratory data on third-generation cephalosporin-resistant Enterobacterales (3GCR-E) from Oslo University Hospital (OUH). We described the incidence of MDROs and resistance proportion in Enterobacterales from 6 months prior to the person’s first cancer diagnosis and up to 3 years after. The cohort included 322,005 persons, of which 0.3% (878) were diagnosed with notifiable MDROs. Peak incidence rates per 100,000 person-years were 60.9 for MRSA, 97.2 for V/LRE, and 6.8 for CP-GNB. The proportion of 3GCR-E in Enterobacterales in blood or urine cultures at OUH was 6% (746/12,534). Despite overall low MDRO incidence, there was an unfavourable trend in the incidence and resistance proportion of Gram-negative bacteria. To address this, there is a need for effective infection control and surveillance. Our study demonstrated the feasibility of expanding the surveillance of MDROs and at-risk populations through the linkage of existing laboratory and register data.

**Supplementary Information:**

The online version contains supplementary material available at 10.1007/s10096-023-04698-3.

## Introduction

Globally, resistance to antimicrobial drugs is increasing in disease-causing bacteria, leading to a higher disease burden [1]. Norway is a low-prevalence setting for resistant bacteria; however, the incidence of multidrug-resistant organisms (MDROs) increased up to 2017 [2]. More than half of the disease burden caused by antimicrobial-resistant pathogens in Europe was associated with healthcare use [3]. Antimicrobial resistance is now threatening to stop and even reverse recent advances in the treatment of one of the largest populations in healthcare, persons with cancer, by making standard treatments such as aggressive chemotherapeutic courses, major surgery, and transplantation unsafe due to the risk of untreatable infectious complications [4]. As cancer patients are often immunocompromised, either due to their disease or treatments, they are assumed to be more susceptible to infections and at greater risk of unfavourable outcomes when infected [5].

As antimicrobial resistance is threatening to disrupt the care of susceptible populations like persons with cancer, there is a need to ensure special consideration of these populations in infectious disease surveillance, both to evaluate the risks and inform prevention efforts. In Norway, methicillin-resistant *Staphylococcus aureus* (MRSA), vancomycin- and/or linezolid-resistant enterococci (V/LRE), and carbapenemase-producing Gram-negative bacilli (CP-GNB) are the main MDROs which are notifiable upon finding to the Norwegian Surveillance System for Communicable Diseases (MSIS) [6]. The notifiable resistant microbes represent the most clinically important resistant bacteria along with third-generation cephalosporin-resistant Enterobacterales (3GCR-E), because of their relative impact [3]. The latter, however, is not notifiable, so data on the incidence of these microbes must be inferred from routinely collected local health data, or deduced from the Norwegian Surveillance System for Antimicrobial Resistance (NORM) [7].

This study aimed to use existing laboratory and register data to describe the incidence or resistance proportion of MDROs in persons diagnosed with cancer in Norway, from 2008 to 2018. Infection and/or colonisation of the notifiable MDROs MRSA, V/LRE, and CP-GNB was described in a national cohort, where data are retrieved from central health registers. In a sub-cohort treated at Oslo University Hospital (OUH), the resistance proportion of 3GCR-E was described.

## Methods

This study is a register-based cohort study describing the surveillance of four categories of MDROs among persons diagnosed with cancer in Norway from 2008 to 2018. The study was designed in accordance with the framework for descriptive epidemiological studies proposed by Lesko et al. (Supplementary Information S7) [8]. It was approved by the Regional Ethics Committee for South-Eastern Norway and recommended by the Data Protection Officer at OUH following a data protection impact assessment.

### Data

#### Data sources and cohort

Data for this study was received from the Cancer Registry of Norway (CRN) and linked to data from the Norwegian Surveillance System for Communicable Diseases (MSIS). The data from CRN was also linked to data from microbiological laboratory systems at OUH. We identified which persons were healthcare workers between 2015 and 2018 by linking data to employment data from Statistics Norway (SSB) in accordance with previously published occupation and industry codes [9]. All data sources were linked on an individual level, establishing a cohort of all persons diagnosed with cancer in Norway from 2008 to 2018. Refusal of treatment—whether therapeutic or palliative—is exceedingly rare in Norway. Thus, those diagnosed within the specified time frame are also likely to have undergone some form of treatment, which should be considered part of their total exposure. Linkage was performed using a hashed, pseudonymised running number based on the national identity number given to each Norwegian citizen or resident, either upon birth or when granting permanent/temporary residency in Norway.

#### Case definition

Cases in our study were defined as persons who were diagnosed with cancer in Norway of any age and gender, with a Norwegian nationality or a permanent or temporary residency in Norway, from 1 January 2008 to 31 December 2018 and registered in the CRN, and who were either registered with a notifiable MDRO in the national register MSIS or where third-generation cephalosporin-resistant Enterobacterales were found in their urine or blood with data on susceptibility to either of the third-generation cephalosporins ceftazidime or cefotaxime at the OUH microbiological laboratory. A flowchart of the collation of the study cohort can be found in Supplementary Information S1. Microbes may be notifiable to MSIS irrespective of their species and which material they are found in as long as they meet the criteria for resistant microbes. Their respective gene expressions and/or minimum inhibitory concentrations to specific drugs can be found in the Supplementary Information S2.

#### Outcomes

In the national cohort, we described the incidence of MDROs that were nationally notifiable during the observation period (CP-GNB were not notifiable before 2012). In the OUH sub-cohort, we described the proportion of Enterobacterales that were resistant to third-generation cephalosporins. To fully capture exposures related to their cancer disease, we set the observation time to start from 6 months prior to and up to 3 years after the persons’ first cancer diagnosis in the study period. This means the observation period could go back to 1 July 2007 and extend to 31 December 2021. All 3GCR-E were detected in blood or urine samples, thus representing infections, ensuring comparability over time regardless of changing screening routines or outbreak management. This also ensured comparability with national surveillance numbers. For notifiable resistant bacteria, infection or colonisation is determined by the reporting clinician. If the infection status was not reported, we defined the infection status with an algorithm that used information on clinical presentation, test indication, or test material (Supplementary Information S3). If there was more than one notification for a person in the period, we described the infection closest to the time of cancer diagnosis, and if no infection was notified, we described the colonisation closest to the time of cancer diagnosis. Few persons were registered with more than one episode (Supplementary Information S1). The same priority was applied to determine the inclusion of 3GCR-E in the OUH sub-cohort.

#### Covariates

The cancer diagnoses were categorised into 12 types based on their localisation within the body, following the grouping of chapters in ICD-10. In addition to metastases where the primary site was unknown, or cancers where the reporting institution had failed to or had not been able to specify the primary site, the category of other cancers included cancers of the bone and cartilage, mesothelia, and soft tissues. These were grouped together due to the low incidence of such cancers. Basal cell carcinomas were not included. Other covariates in this study included histopathological stage of the cancer (local, regional, or distant) at the time of diagnosis as reported to the CRN by the treating physician. We also described whether the person experienced a new cancer at the same site, new cancer at a different site, or a regional or distant metastasis that was not present at the first diagnosis. Additionally, the date of birth, assigned sex at birth, and the municipality of residence at the time of cancer diagnosis were collected from CRN. For the notifiable MDROs, we described whether the person was notified as infected within Norway or abroad.

### Statistical analysis

We present descriptive data for each covariate for person with and without an MDRO, and the infection status and place of transmission for persons with an MDRO. For persons in which Enterobacterales was found in the OUH sub-cohort, we described the proportion resistant to third-generation cephalosporins. We graphically displayed the incidence rates of infection or colonisation with notifiable MDROs by 100,000 person-years across the study period. For 3GCR-E, we graphically displayed the resistance proportion by calendar year. The full species distribution found in blood or urine at OUH can be found in the Supplementary Information. Density estimates for when MDROs were found in the observation time were calculated. We estimated the incidence rate ratios (IRRs) for infection or colonisation with either MRSA or V/LRE by different cancer types by fitting a robust Poisson regression model with observation time as an offset to the incidence of MDRO infection or colonisation, using gastrointestinal cancers as the reference category [10]. The model was standardised by age and sex by fitting linear terms for these covariates. We did not model CP-GNB due to the low number of notifications in the period. As a secondary analysis, we also used the same modelling approach to test the association between MDROs and occupation as registered during the observation time, as it may be hypothesised that healthcare workers are more exposed to MDROs as these microbes are more often found in the healthcare setting [3]. To investigate the trends of the incidence rates and resistance proportions, Poisson regression models with person-years as an offset were also fitted to the aggregated incidence with a linear term for calendar year.

The register data used in this study can be accessed through an application with the necessary ethical approvals to Helsedataservice [11]. All analyses were performed in R version 4.1.3 using RStudio [12, 13]. The scripts used for analysis have been made publicly accessible on GitHub [14].

## Results

### Study population and cancer

We included 322,005 persons diagnosed with cancer between 2008 and 2018 in Norway, of which 170,378 (47%) were female (Table 1). The median age of the persons was 69 years at diagnosis of cancer (IQR, 59–78 years). Gastrointestinal cancers (incl. colorectal, stomach, pancreas, and liver) were the most common cancer type in both sexes (64,842, 20%). The next most common cancer overall were cancers of the male reproductive system (54,064, 17%).

**Table 1:** Characteristics of persons diagnosed with cancer in Norway from 2008 to 2018 at the time of their first cancer diagnosis, stratified by whether they were reported with multidrug-resistant organisms (MDROs) from 6 months prior to or up to 3 years after cancer diagnosis. MDROs include methicillin-resistant *Staphylococcus aureus* (MRSA), vancomycin- and/or linezolid-resistant enterococci (V/LRE), and carbapenemase-producing Gram-negative bacteria (CP-GNB). *All variables are presented as counts with percentages by row, except for age in years which is presented with median and interquartile range

	Overall			Resistant notifiable microbe		
	Overall, *N* = 322,005	No notifiable microbe, *n* = 321,127	Notifiable microbe, *n* = 878	MRSA, *n* = 458	V/LRE, *n* = 396	CP-GNB, *n* = 24
Age in years*	68 (59, 78)	68 (59, 78)	69 (58, 78)	68 (56, 79)	70 (60, 78)	67 (51, 70)
Sex						
F	151,627 (100.00%)	151,250 (99.75%)	377 (0.25%)	201	165	11
M	170,378 (100.00%)	169,877 (99.71%)	501 (0.29%)	257	231	13
Residency in Oslo						
Oslo	31,917 (100.00%)	31,802 (99.64%)	115 (0.36%)	91	18	6
Other residency	290,088 (100.00%)	289,325 (99.74%)	763 (0.26%)	367	378	18
Country of birth						
Norway	299,265 (100.00%)	298,530 (99.75%)	735 (0.25%)	352	367	16
Other (or missing)	22,740 (100.00%)	22,597 (99.37%)	143 (0.63%)	106	29	8
Type of cancer						
Breast	35,121 (100.00%)	35,066 (99.84%)	55 (0.16%)	47	8	0
Eye, brain, and central nervous system	12,024 (100.00%)	11,999 (99.79%)	25 (0.21%)	19	< 5	< 5
Female reproductive system	17,577 (100.00%)	17,552 (99.86%)	25 (0.14%)	16	7	< 5
Gastrointestinal	64,842 (100.00%)	64,651 (99.71%)	191 (0.29%)	88	96	7
Lymphoid or haematopoietic	28,033 (100.00%)	27,872 (99.43%)	161 (0.57%)	48	107	6
Male reproductive system	54,064 (100.00%)	53,978 (99.84%)	86 (0.16%)	59	26	< 5
Oral	5750 (100.00%)	5728 (99.62%)	22 (0.38%)	14	7	< 5
Others or unknown	6428 (100.00%)	6397 (99.52%)	31 (0.48%)	15	16	0
Respiratory and intrathoracic	31,485 (100.00%)	31,369 (99.63%)	116 (0.37%)	38	73	5
Skin	37,666 (100.00%)	37,586 (99.79%)	80 (0.21%)	64	16	0
Thyroid and endocrine system	5752 (100.00%)	5738 (99.76%)	14 (0.24%)	12	< 5	0
Urinary tract	23,263 (100.00%)	23,191 (99.69%)	72 (0.31%)	38	33	< 5
Cancer dissemination						
Distant	47,000 (100.00%)	46,859 (99.70%)	141 (0.30%)	56	80	5
Localised	137,189 (100.00%)	136,911 (99.80%)	278 (0.20%)	182	91	5
Regional	70,394 (100.00%)	70,201 (99.73%)	193 (0.27%)	116	72	5
Unknown	67,422 (100.00%)	67,156 (99.61%)	266 (0.39%)	104	153	9
Cancer progression						
New cancer, new site	18,973 (100.00%)	18,923 (99.74%)	50 (0.26%)	23	26	< 5
New cancer, same site	4261 (100.00%)	4256 (99.88%)	5 (0.12%)	< 5	< 5	0
New metastasis	7730 (100.00%)	7712 (99.77%)	18 (0.23%)	10	7	< 5
No new cancer	291,041 (100.00%)	290,236 (99.72%)	805 (0.28%)	421	362	22
Surgery reported						
No	120,797 (100.00%)	120,375 (99.65%)	422 (0.35%)	181	229	12
Yes	190,420 (100.00%)	189,999 (99.78%)	421 (0.22%)	265	145	11
Missing	10,788 (100.00%)	10,753 (99.68%)	35 (0.32%)	12	22	< 5
Radiotherapy reported						
No	221,200 (100.00%)	220,629 (99.74%)	571 (0.26%)	298	257	16
Yes	77,218 (100.00%)	76,993 (99.71%)	225 (0.29%)	115	105	5
Missing	23,587 (100.00%)	23,505 (99.65%)	82 (0.35%)	45	34	< 5

### MDROs

**Fig. 1:**
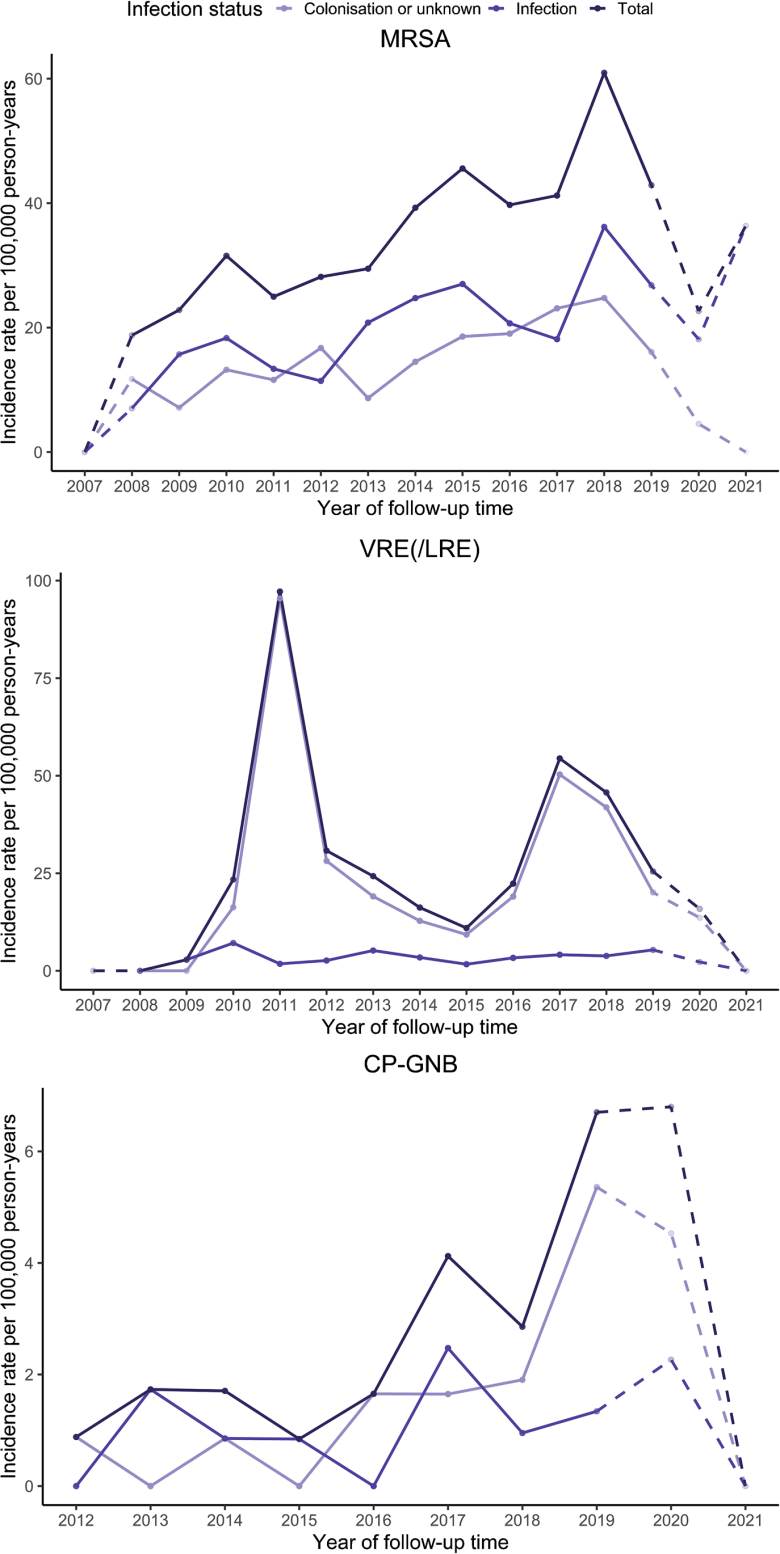
Density estimates for the finding of multidrug-resistant organisms (MDROs) by time since cancer diagnosis 6 months prior to or up to 3 years after the first cancer diagnosis of persons in Norway, from 2008 to 2018. MDROs include methicillin-resistant *Staphylococcus aureus* (MRSA), vancomycin- and/or linezolid-resistant enterococci (V/LRE), carbapenemase-producing Gram-negative bacilli (CP-GNB), and third-generation cephalosporin-resistant Enterobacterales (3GCR-E)

In 878 (0.3%) of the persons, MRSA, V/LRE, or CP-GNB was reported. Of those, 458 (52%) were MRSA, 396 (45%) were V/LRE, and 24 (3%) were CP-GNB. Among the resistant enterococci, 10 were linezolid-resistant, and fewer than 5 were resistant to both vancomycin and linezolid. The highest proportion of MDROs were found in persons with cancer of the lymphoid or haematopoietic tissues (161/28,033, 0.6%), and the lowest proportion were found in those diagnosed with cancers of the female reproductive system (25/17,577, 0.1%). MDROs were most often found in between the time of cancer diagnosis and up to 100 days after cancer diagnosis, except for CP-GNB, which were found in a bimodal pattern with peaks directly after cancer diagnosis and 2 years after cancer diagnosis (Fig. 1).

### Incidence and characteristics of notifiable MDROs

**Fig. 2:**
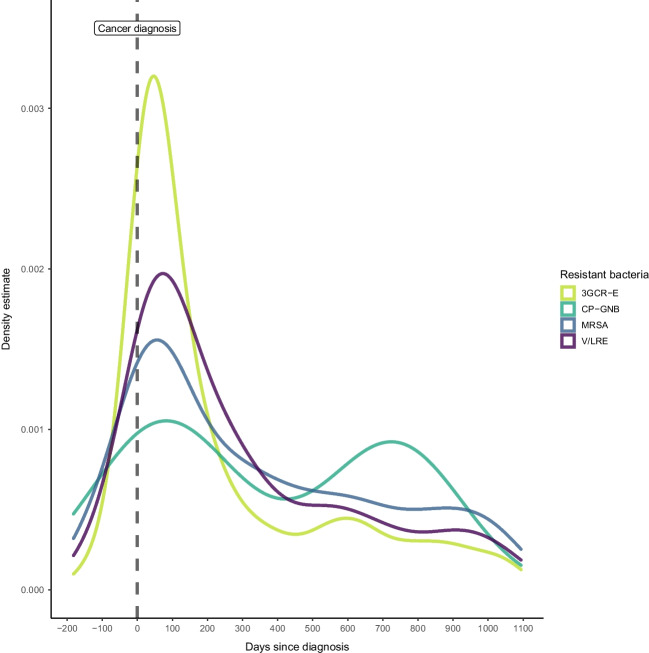
Incidence rates of infection, colonisation, and total of multidrug-resistant organisms (MDROs) by person-years, found in persons 6 months prior to or up to 3 years after their first cancer diagnosis in Norway from 2008 to 2018. Dashed lines represent observation time outside of the inclusion period, with diminishing person-years. MDROs include methicillin-resistant *Staphylococcus aureus* (MRSA), vancomycin- and/or linezolid-resistant enterococci (VRE/LRE), and carbapenemase-producing Gram-negative bacilli (CP-GNB)

Of all 878 notifications, 317 (36%) notified of an infection and 497 (57%) were colonisation. The algorithm to define colonisation and the resulting proportions can be found in Supplementary Information S3 and S4. Colonisation was most frequently reported for V/LRE (73%, 291/396), while there were no major differences in the proportion of colonisation or place of transmission in respect to the time from cancer diagnosis for any of the MDROs. Of the three resistant microbes, CP-GNB was most reported with a foreign country as the place of infection (12/24, 50%). The incidence rates of MRSA rose steadily until 2018 when it peaked at 60.9 per 100,000 person-years and then decreased with a *p*-value for the linear trend across the whole period of < 0.001 (Fig. 2). Two distinct peaks were observed for V/LRE, the first one in 2011 with a total of 97.2 per 100,000 person-years and the second one in 2017 with a total of 54.4 per 100,000 person-years with a *p*-value for the linear trend of 0.6. CP-GNB rose until it peaked in 2020 at 6.8 per 100,000 person-years, with no notifications in 2021 and a *p*-value for the linear trend of 0.009. The total amount of person-years included by year in the study period can be found in Supplementary Information S5.

**Fig. 3:**
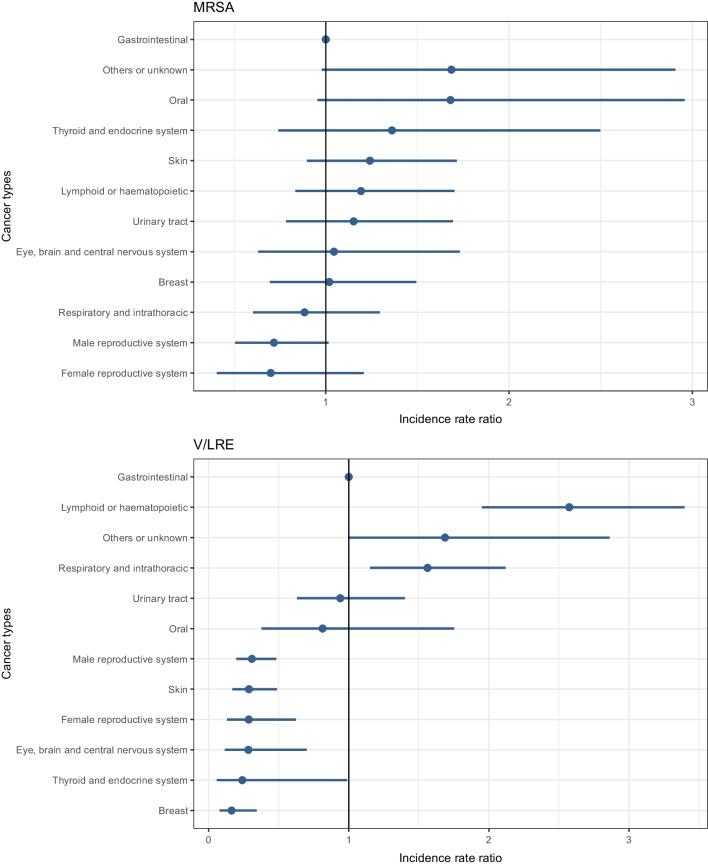
Age- and sex-standardised incidence rate ratios (IRR) of infection/colonisation with either methicillin-resistant *Staphylococcus aureus* (MRSA) or vancomycin- and/or linezolid-resistant enterococci (V/LRE) in persons 6 months prior to or up to 3 years after their first cancer diagnosis in Norway, from 2008 to 2018, by different cancer types

For the IRR for MRSA, there were no differences observed between different types of cancers compared to gastrointestinal cancers. The IRR for V/LRE, however, was highest in persons with cancers of the lymphoid or haematopoietic tissues with an IRR of 2.57 (95% CI, 1.95–3.40) and lowest for breast cancer with an IRR of 0.16 (95% CI, 0.08–0.34) compared to gastrointestinal cancers (Fig. 3).

There was no evidence of a difference in MDRO incidence in healthcare workers compared to other persons diagnosed with cancer between 2015 and 2018 (*p* = 0.36).

### Resistance proportion and characteristics of 3GCR-E

**Fig. 4:**
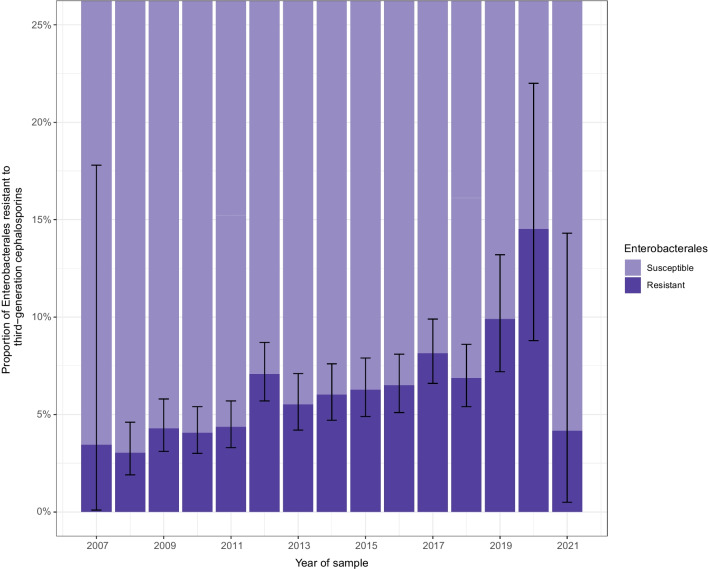
The proportion of Enterobacterales resistant to third-generation cephalosporins found in the blood or urine of persons diagnosed with or treated for cancer at Oslo University Hospital by year the sample was taken, found from 6 months prior to or up to 3 years after the first cancer diagnosis from 2008 to 2018

In the OUH sub-cohort, Enterobacterales were diagnosed from the blood or urine of 12,534 persons, of which 746 (6%) were 3GCR (Table 2). Persons in which 3GCR-E infections were found in blood or urine were slightly younger, 65 years (interquartile range, 55–73 years) vs 69 years (interquartile range, 60–78 years) and more often female (6852/12,534, 55%). 3GCR-E was more common in persons born outside Norway (178/1184, 13%) compared to those born within Norway (568/11172, 5%). It was also most common in those diagnosed with cancers of the lymphoid or haematopoietic tissues (116/1230, 9%) compared to other cancers. The most common species of Enterobacterales cultured was *Escherichia coli* (7704/12,534, 61%), while *Klebsiella pneumoniae* represented 8% (1050/12,543) of all Enterobacterales. Resistance to third-generation cephalosporins was found in 5.3% (405/7704) of *Escherichia coli* and 8.0% (84/1050) of *Klebsiella pneumoniae*. The proportion of resistance to third-generation cephalosporins was lowest among all Enterobacterales in 2008 (3.0%, 95% CI 1.9–4.6%), and highest in 2020 (14.5%, 95% CI 8.8–22.0%) with a *p*-value for the linear trend of < 0.001 (Fig. 4). The full species distribution found in blood or urine of persons diagnosed or treated at OUH can be found in Supplementary Information S6.

**Table 2:** Characteristics of persons diagnosed with or treated for cancer at Oslo University Hospital from 2008 to 2018, in whom Enterobacterales were found in blood or urine from 6 months prior to or up to 3 years after first cancer diagnosis. The table is stratified by whether they were found to have a third-generation cephalosporin-resistant Enterobacterales (3GCR-E) or third-generation cephalosporin-susceptible Enterobacterales (3GCS-E). *All variables are presented as counts with percentages by row, except for age which is presented with median and interquartile range

	Overall, *N* = 12,534	3GCS-E, *n* = 11,788	3GCR-E, *n* = 746
Age in years*	69 (60, 78)	69 (60, 78)	65 (55, 73)
Sex			
F	6852	6506 (94.95%)	346 (5.05%)
M	5682	5282 (92.96%)	400 (7.04%)
Oslo			
Oslo	6687	6300 (94.21%)	387 (5.79%)
Other residency	5847	5488 (93.86%)	359 (6.14%)
Country of birth			
Norway	11,172	10,604 (94.92%)	568 (5.08%)
Other (or missing)	1362	1184 (86.93%)	178 (13.07%)
Species			
*Citrobacter* spp.	288	256 (88.89%)	32 (11.11%)
*Enterobacter* cloacae	480	387 (80.62%)	93 (19.38%)
*Enterobacter* spp.	469	408 (86.99%)	61 (13.01%)
*Escherichia coli*	7704	7299 (94.74%)	405 (5.26%)
*Klebsiella pneumoniae*	1050	966 (92.00%)	84 (8.00%)
*Klebsiella* spp.	1192	1155 (96.90%)	37 (3.10%)
*Morganella* spp.	119	110 (92.44%)	9 (7.56%)
*Proteus* spp.	897	886 (98.77%)	11 (1.23%)
*Providencia* spp.	8	8 (100.00%)	0 (0.00%)
*Serratia* spp.	251	243 (96.81%)	8 (3.19%)
Other findings	76	70 (92.11%)	6 (7.89%)
Type of cancer			
Breast	810	778 (96.05%)	32 (3.95%)
Eye, brain, and central nervous system	398	366 (91.96%)	32 (8.04%)
Female reproductive system	1398	1342 (95.99%)	56 (4.01%)
Gastrointestinal	3402	3178 (93.42%)	224 (6.58%)
Lymphoid or haematopoietic	1230	1114 (90.57%)	116 (9.43%)
Male reproductive system	1405	1339 (95.30%)	66 (4.70%)
Oral	284	258 (90.85%)	26 (9.15%)
Others or unknown	360	340 (94.44%)	20 (5.56%)
Respiratory and intrathoracic	1202	1128 (93.84%)	74 (6.16%)
Skin	937	899 (95.94%)	38 (4.06%)
Thyroid and endocrine system	127	122 (96.06%)	5 (3.94%)
Urinary tract	981	924 (94.19%)	57 (5.81%)
Cancer dissemination			
Distant	2,296	2150 (93.64%)	146 (6.36%)
Localised	3988	3783 (94.86%)	205 (5.14%)
Regional	3548	3360 (94.70%)	188 (5.30%)
Unknown	2702	2495 (92.34%)	207 (7.66%)
Cancer progression			
New cancer, new site	918	856 (93.25%)	62 (6.75%)
New cancer, same site	142	137 (96.48%)	5 (3.52%)
New metastasis	363	346 (95.32%)	17 (4.68%)
No new cancer	11,111	10,449 (94.04%)	662 (5.96%)
Surgery reported			
No	4431	4140 (93.43%)	291 (6.57%)
Yes	7710	7283 (94.46%)	427 (5.54%)
Missing	393	365 (92.88%)	28 (7.12%)
Radiotherapy reported			
No	8175	7698 (94.17%)	477 (5.83%)
Yes	3578	3374 (94.30%)	204 (5.70%)
Missing	781	716 (91.68%)	65 (8.32%)

## Discussion

In this register-based cohort study describing the surveillance of MDROs among persons diagnosed with cancer, we found that 0.3% were registered with MRSA, V/LRE, or CP-GNB. In a sub-cohort of persons diagnosed or treated at OUH, we found that 6% of Enterobacterales cultured from the urine or blood of persons with cancer were resistant to third-generation cephalosporins. We found that persons with cancers of the lymphoid or haematopoietic tissues had the highest incidence of MDRO infection/colonisation and the highest proportion of resistance in Enterobacterales infection/colonisation compared to persons with other types of cancer.

In our work, we have demonstrated how it is possible to expand the register-based surveillance at the national level to include both potentially susceptible patient populations using existing register data and organisms like 3GCR-E using the richer microbiological laboratory data from a hospital. 3GCR-E is not nationally notifiable in Norway, yet is responsible for a large proportion of the total disease burden of AMR [3]. As the capture and linkage of existing regional laboratory data and national register data may be automated, it may be possible to expand surveillance without increasing the resource requirements [15]. Previous work has shown how it is possible to expand surveillance to detect outbreaks and clusters in the healthcare setting through the use of automated algorithms to capture and link existing register data [16, 17]. The challenges posed to such an expansion of surveillance include the need for standardised protocols to capture heterogenous laboratory data from multiple sources and ensure the privacy of the patient [15].

The proportion of colonisation and the development in the trends of notifications were similar to a previous study of the epidemiology of MRSA, V/LRE, and CP-GNB in the whole population of Norway, 2006–2017 [2]. Elstrøm and colleagues concluded that incidence rates were increasing up until 2017. It is uncertain whether this trend is carried forward due to disruptions from the pandemic. Compared to the incidence rates of Elstrøm et al. and publicly available surveillance data found when selecting the same categories of MDROs and years on the website of the Norwegian Institute of Public Health [18], our cohort of persons diagnosed with cancer has more than three times the rate of V/LRE, but only about a fourth the rate of MRSA [2, 7]. The most likely reasons for these discrepancies could be that V/LRE in Norway has predominantly been associated with large outbreaks in healthcare institutions with extensive contact tracing and screening [2, 19–21].

One could hypothesise that the higher V/LRE incidence was not only a consequence of heightened susceptibility to infection, but also of the time spent in healthcare. This may have led to both an increased probability of being implicated in ongoing nosocomial outbreaks, and as such of being tested and found positive, and a selection for V/LRE colonisation in patients who receive frequent antibiotic treatment, in particular those with cancers of the gastrointestinal tract, as supported by the incidence rate ratios [22]. The higher rates of discovered V/LRE colonisation in persons with cancers of the lymphoid and haematopoietic tissues and persons with cancers of unknown origin (often metastasised at the time of diagnosis) are in accordance with this hypothesis, due to their frequently prolonged hospital stays. The increased finding of resistant bacteria directly after cancer diagnosis may also reflect screening on admittance or prior to procedures, rather than acquisition at that time. We also tested a secondary hypothesis that persons with cancer and who were healthcare workers were more often infected or colonised with resistant bacteria than persons with cancer and other occupations, as they were potentially more exposed to the bacterial flora in the healthcare setting but found no indication of this. As V/LRE colonisation is a risk factor of the development of V/LRE bacteraemia, and many persons with cancer are vulnerable to infections, the increased rate of colonisation in this group is concerning and underlines the importance of reducing the prevalence of such bacteria in the healthcare setting [23, 24].

MRSA, on the other hand, has previously been shown to be predominantly acquired in the community or associated with travel from abroad [2, 21, 25]. If people living with cancer defer from travelling and shield themselves, either consciously to avoid complicating infections during cancer treatment or because they are too ill to participate in the community, their exposure to situations associated with MRSA (and CP-GNB) acquisition may decrease. The fact that a lower proportion of notifications were reported with a place of *transmission* outside of Norway for persons with cancer compared to the general population supports that persons with cancer defer from travelling to some extent [18, 25]. Although there are few studies on the epidemiology of resistant Gram-positive bacteria in national cancer cohorts, compared to studies using clinical data, the incidence rates of in our cohort were low [26].

We found that although Enterobacterales found in the blood or urine of persons with cancer at OUH have a higher proportion of resistance to the third-generation cephalosporins cefotaxime and ceftazidime than the national average of *Klebsiella* spp. and *Escherichia* spp. found in blood or urine as reported in the NORM-NORM/VET surveillance, the resistance proportion was similar to the mean of the whole hospital [7, 27]. This could be because OUH is Norway’s only tertiary hospital, and only specialist cancer hospital and transplantation centre. This means that the hospital admits patients with higher or more complex comorbidity than other regional hospitals and that patients may be transferred to OUH after lengthy stays in other institutions, which may increase the risk of antimicrobial resistance. Furthermore, we found a higher resistance proportion of 3GCR-E in patients with cancers of the lymphoid or haematopoietic tissues, which may reflect that the nature of the immunosuppression or specific antibiotic regimens given to these patients selects for this type of antimicrobial resistance. While resistance to third-generation cephalosporins appears to be steadily increasing in Norway, we found a lower proportion of such resistance overall in our study cohort compared to the averages in the European general population found in the EARS-NET reports [1, 7, 28].

CP-GNB was also increasing throughout the study period. However, there were no reported CP-GNB in our study cohort in 2021, during the COVID-19 pandemic. This may not have been an effect of the pandemic alone, but also due to the gradual depletion of observation time in 2021, as only persons diagnosed with cancer in 2018 could be followed into 2021. It could also be related to the limited travel during the pandemic, as the sporadic cases of CP-GNP in Norway are commonly associated with import, demonstrated by the fact that half of the CP-GNB in our study were reported to be acquired abroad. This association was also reflected by the sharp decline in CP-GNB notifications during the COVID-19 pandemic in the general population [18]. Other countries have reported an increase in typically healthcare-associated or emerging carbapenem-resistant Gram-negative bacteria during the pandemic, like *Acinetobacter baumannii* [29, 30]. However, a study performed in an endemic setting indicated a decline in carbapenem-resistant *Klebsiella pneumoniae* infections, potentially as a result of COVID-19 prevention measures [31]. The potential of endemicity may be due to both successful clones and the potential for rapid, horizontal transferring of plasmid-mediated resistance such as carbapenemases and extended-spectrum beta-lactamases, with several examples globally [32]. With this potential in mind, prevention of resistant Gram-negative bacteria with mobile genetic elements should be intensified in the coming years, particularly with the reopening of international travel. When such bacteria become widespread in the healthcare setting, it may prove impossible to reverse the situation.

The strengths of our study are that we present notifications of resistant microbes in a nationwide and complete cohort of persons diagnosed with cancer. For persons diagnosed or treated at OUH, we have complete microbiological data, allowing us to include bacteria that are not nationally notifiable. In addition, we retrieved occupational data, allowing us to test a secondary hypothesis. However, we could not know whether persons with cancer were actively working as healthcare workers in the full observation time, or if they quit or were on extended sick leave. It is also important to consider our open cohort structure when interpreting the data, in which observation time gradually accumulates at the beginning and gradually depletes towards the end of the study period. The incidence rates in those years are therefore calculated in a few persons, meaning interpretation should be done with care. We have also only included the infectious diagnosis closest in time to the cancer diagnosis, as the same microbe was often found multiple times. Further, we were not able to present results and discuss the clinical outcomes of colonisation or infection with resistant microbes, as we did not retrieve clinical data. In the OUH sub-cohort, we did not have a complete denominator allowing us to calculate the incidence rate of Enterobacterales among all persons with cancer who had any part of their diagnostic workup or treatment at the hospital. Although exceedingly rare before that year, CP-GNB were not notifiable prior to 2012, leading to a possible underestimation early in the study period. Finally, the data on resistant microbes are only representative of the tests performed and were thus sensitive to changing guidelines and routines for screening, or special situations like ongoing outbreak investigations.

In a cohort of persons diagnosed with cancer in Norway between 2008 and 2018, we found that the incidence rate of resistant Gram-positive bacteria has not increased in recent years although we observed more of the often healthcare-associated V/LRE and less of the often community-associated MRSA compared to previously published data on the general population. It may seem like infection prevention and control measures aimed at containing (resistant) Gram-positives are succeeding. However, we observed an unfavourable trend in both incidence rates and resistance proportions of Gram-negative bacteria. Furthermore, we found both the highest incidence rates and resistance proportions in patients with cancers of the lymphoid or haematopoietic tissues, possibly reflecting specific healthcare exposures like prolonged hospitalisation or antibiotic treatment. Regardless of what is causing the increase, infection prevention efforts aimed at controlling Gram-negative bacteria with the potential for acquiring extensive drug resistance should be intensified to protect potentially susceptible groups like persons living with cancer. We have demonstrated that it is possible to expand the surveillance of infectious diseases at the national level to both include such potentially susceptible populations and non-notifiable pathogens by combining existing data sources.

### Supplementary Information

Below is the link to the electronic supplementary material.

Supplementary file1 (DOCX 224 KB)

## Data Availability

The data used in this study may be accessed through an application to Helsedataservice following necessary ethical approvals.
