# Supplementary material for: The epidemiology of multidrug-resistant organisms in persons diagnosed with cancer in Norway, 2008–2018: expanding surveillance using existing laboratory and register data
Source: Eur J Clin Microbiol Infect Dis. 2023 Nov 18;43(1):121–32. doi: 10.1007/s10096-023-04698-3 (PMC10774199; doi:10.1007/s10096-023-04698-3)
Supplement: Supplementary file 1 — Supplementary file1 (DOCX 224 KB) [file 10096_2023_4698_MOESM1_ESM.docx]

## Supplementary Information

This is the supplementary information to the article “The epidemiology of multidrug-resistant organisms in persons diagnosed with cancer in Norway, 2008-2018: expanding surveillance using existing laboratory and register data” in the European Journal of Clinical Microbiology and Infectious Diseases by authors Anders Skyrud Danielsen Petter Elstrøm, Hanne-Merete Eriksen-Volle, Solveig Hofvind, David W. Eyre, Oliver Kacelnik, and Jørgen Vildershøj Bjørnholt.

Corresponding author: Anders Skyrud Danielsen ([andersskyrud.danielsen@fhi.no](mailto:andersskyrud.danielsen@fhi.no)).


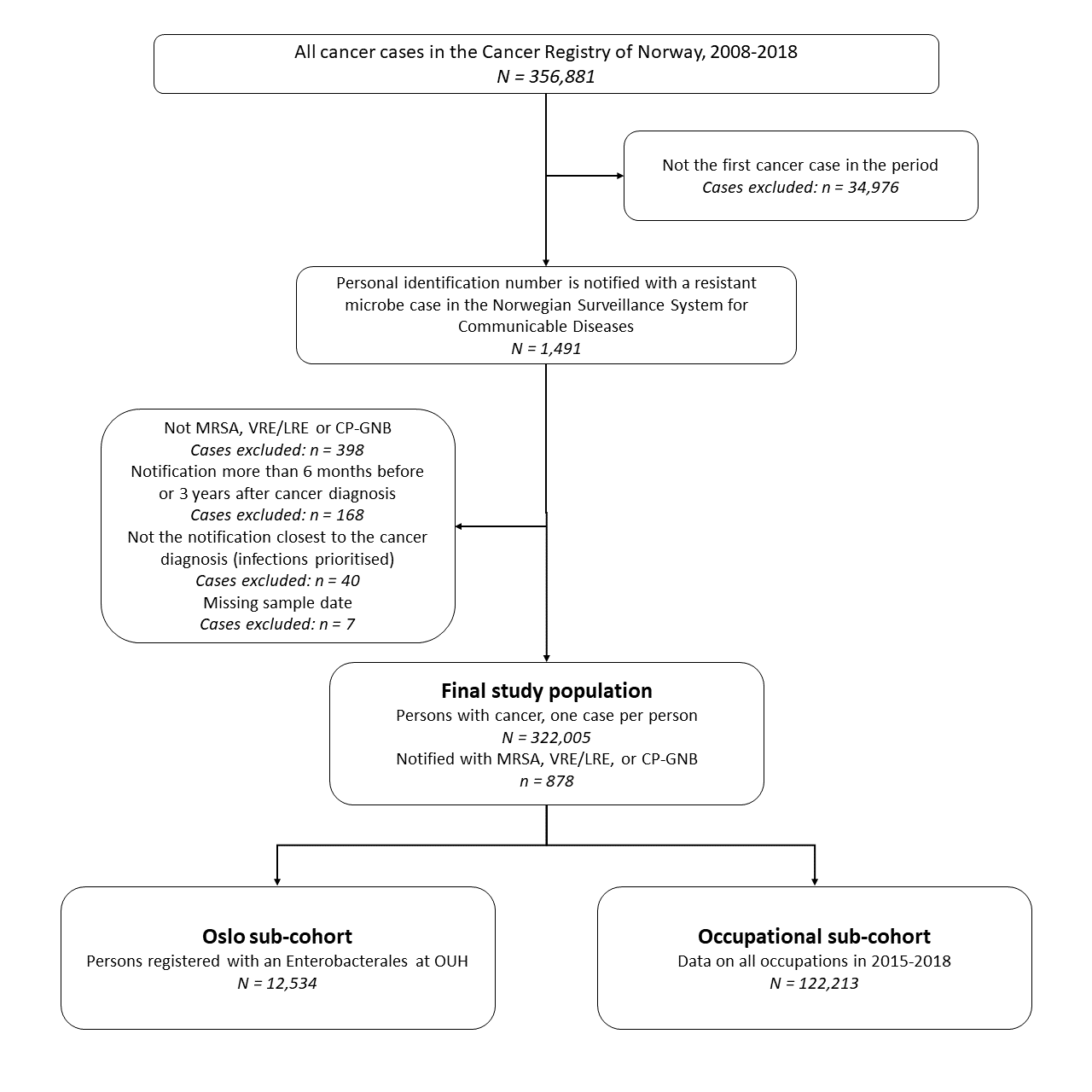


Figure S1: Flowchart of the study population, from all cancer cases to one case per person.

Supplementary Information S2. Case definitions of multidrug-resistant organisms notifiable to the Norwegian Surveillance System for Communicable Diseases.

- **Methicillin-resistant *Staphylococcus aureus* (MRSA):** All cases of *Staphylococcus aureus* found in any human material, which is resistant against cefoxitin, expressing either the mecA or mecC gene and verified as MRSA by the Norwegian Reference Laboratory for MRSA.
- **Vancomycin- and/or linezolid-resistant enterococci (V/LRE):** All enterococci found in any human material expressing either the vanA or vanB gene and/or with a minimum inhibitory concentration (MIC) for vancomycin of ≥ 4 mg/l and/or with a MIC for linezolid of ≥ 4 mg/l.
- **Carbapenemase-producing Gram-negative bacilli (CP-GNB):** All Enterobacterales or Pseudomonadales found in any human material, which is found to have reduced susceptibility or resistance to meropenem and later verified as carbapenemase-producing by the Norwegian National Advisory Unit on Detection of Antimicrobial Resistance (K-res).

Table S3. The algorithm used for defining infection or colonisation, using information reported by the notifying clinician.

|  |  | **Infection** | **Colonisation** | **Unknown** |
| --- | --- | --- | --- | --- |
| **First priority** | **Infection status** |  |  |  |
|  | Infection | x | - | - |
|  | Colonisation | - | x | - |
|  | Unknown | - | - | x |
| **Second priority** | **Test indication** |  |  |  |
|  | Symptoms or signs | x | - | - |
|  | Routine test, hospital stay | - | x | - |
|  | Routine test, contact with healthcare abroad | - | x | - |
|  | Contact tracing (environmental sample) | - | x | - |
|  | Routine test, unspecified | - | x | - |
|  | Incidental finding | - | - | x |
|  | Other indication | - | - | x |
|  | Routine test, immigrant | - | x | - |
|  | Working with patients or children | - | - | x |
| **Third priority** | **Clinical findings** |  |  |  |
|  | Skin infection, indwelling device (catheter etc) | x | - | - |
|  | Skin abscess | x | - | - |
|  | Asymptomatic | - | x | - |
|  | Pneumonia (LRTI) | x | - | - |
|  | Other | - | - | x |
|  | Urinary tract infection | x | - | - |
|  | Post-operative wound infection | x | - | - |
|  | Sepsis | x | - | - |
|  | Skin/wound infection, unspecified | x | - | - |
|  | URTI | x | - | - |
|  | Wound infection/abscess | x | - | - |
|  | Meningitis/encephalitis | x | - | - |
|  | Gastroenteritis | x | - | - |
|  | Unknown | - | - | x |
|  | Eye infection | x | - | - |
|  | Otitis, external | x | - | - |
|  | Respiratory tract infection, unspecified | x | - | - |
|  | Otitis media | x | - | - |
|  | Gynaecological infection | x | - | - |
| **Fourth priority** | **Material** |  |  |  |
|  | Wound secretion | x | - | - |
|  | Other material | - | - | x |
|  | Pus | x | - | - |
|  | Faeces | - | x | - |
|  | Respiratory tract secretion | - | - | x |
|  | Skin | - | - | x |
|  | Blood | x | - | - |
|  | Urine | - | - | x |
|  | Genital secretion | - | - | x |
|  | BAL | - | - | x |
|  | Unknown material | - | - | x |
|  | Expectorate | - | - | x |
|  | Pleural fluid | x | - | - |
|  | Nasopharynx secretion | - | - | x |
|  | Eye secretion | - | - | x |
|  | Tissue sample/biopsy | x | - | - |
|  | Biopsy | x | - | - |
|  | Spinal fluid | x | - | - |
|  | Abscess content | x | - | - |
|  | Throat secretion | - | - | x |
|  | Ear secretion | - | - | x |
|  | Secretion | - | - | x |
|  | Perineum | - | x | - |

Table S4. Characteristics of multidrug-resistant organisms (MDROs) in persons six months prior to or up to three years after their first cancer diagnosis in Norway from 2008 to 2018, stratified by bacteria. MDROs include methicillin-resistant Staphylococcus aureus (MRSA), vancomycin- and/or linezolid-resistant enterococci (V/LRE), and carbapenemase-producing Gram-negative bacilli (CP-GNB).

|  | **Overall,  N = 878** | **MRSA,  N = 458** | **V/LRE,  N = 396** | **CP-GNB,  N = 24** |
| --- | --- | --- | --- | --- |
| **Infection status** | |  |  |  |
| Colonisation | 497 (57%) | 193 (42%) | 291 (73%) | 13 (54%) |
| Infection | 317 (36%) | 263 (57%) | 44 (11%) | 10 (42%) |
| Unknown/missing** | - | - | - | - |
| **Place of transmission** | |  |  |  |
| Abroad | 91 (10%) | 70 (15%) | 9 (2.3%) | 12 (50%) |
| Norway | 340 (39%) | 178 (39%) | 156 (39%) | 6 (25%) |
| Unknown | 447 (51%) | 210 (46%) | 231 (58%) | 6 (25%) |
| *All variables are presented as counts with percentages by columns.  **Censored. | | | | |

Table S5. Person-years included in the incidence rate calculation in figure 1.

| **Year** | **Person-years** |
| --- | --- |
| 2007 | 15104 |
| 2008 | 42638 |
| 2009 | 70062 |
| 2010 | 98277 |
| 2011 | 112148 |
| 2012 | 113664 |
| 2013 | 115377 |
| 2014 | 117181 |
| 2015 | 118510 |
| 2016 | 120875 |
| 2017 | 121277 |
| 2018 | 105017 |
| 2019 | 74623 |
| 2020 | 44128 |
| 2021 | 13752 |


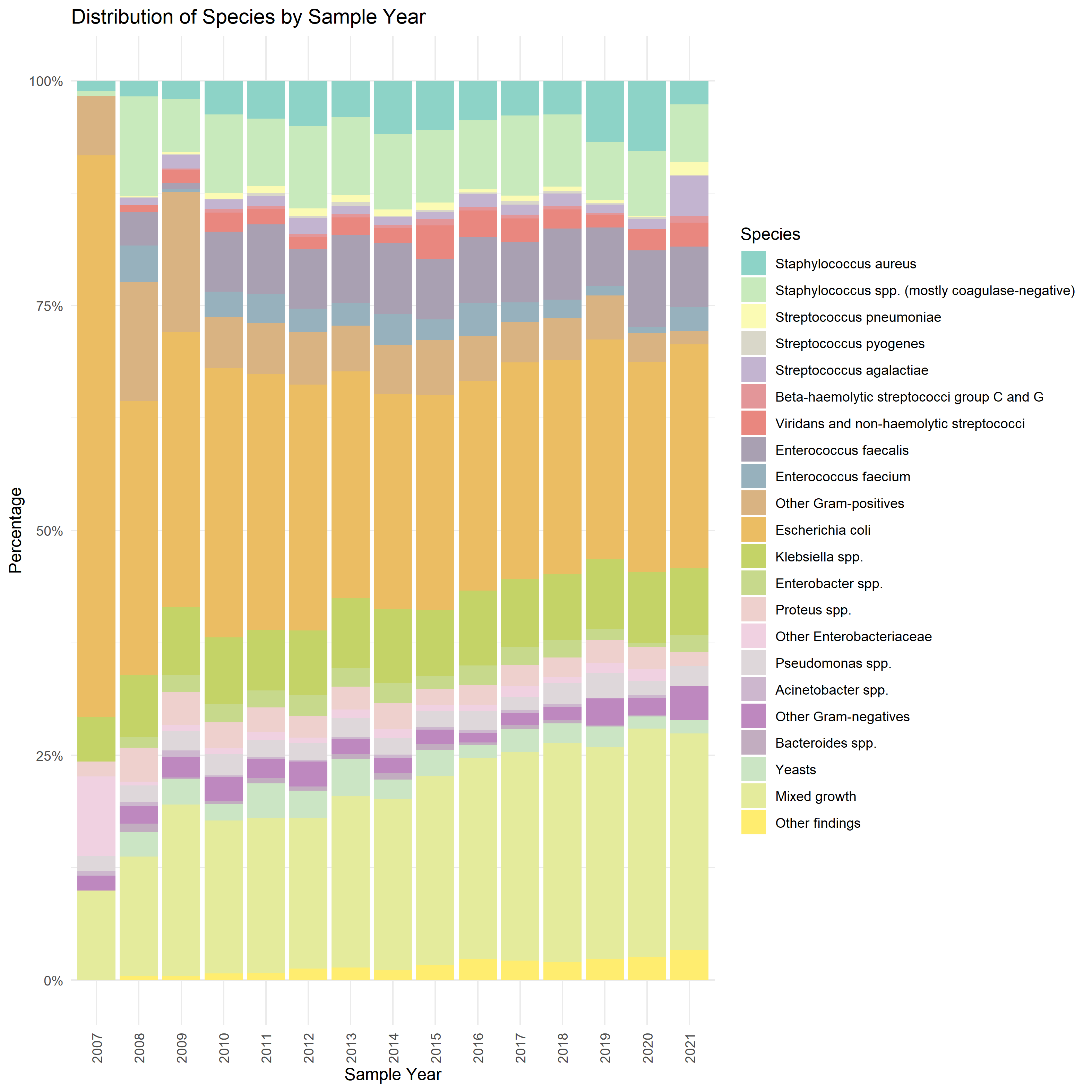


Figure S6: The distribution of all species found in the blood or urine of persons diagnosed with or treated for cancer at Oslo University Hospital by year the sample was taken, found from six months prior to or up to three years after the first cancer diagnosis from 2008 to 2018. The sample was not restricted to one finding per person and included a total of 42,512 findings.

Table S7. Reporting of items in descriptive epidemiological studies.

| **Article Section and Item** | | **Item No.** | **Recommendation(s)** | | **Included** | |
| --- | --- | --- | --- | --- | --- | --- |
| Title and abstract | | 1 | Explicitly state that this is a “descriptive study” in the title or the abstract. | | ✓ | |
|  | | 2 | Summarize the target population and provide an informative and balanced summary of estimated disease occurrence in the abstract. | | ✓ | |
| Introduction | |  |  | |  | |
| Background/rationale | | 3 | State the motivation for the study, including, where relevant, the action that might be informed by the results. | | ✓ | |
| Objectives | | 4 | State the descriptive estimand, explicitly including: | |  | |
|  |  |  | (a) the target population (who would be affected by any decisions made as a result of the study?); | | ✓ | |
|  |  |  | (b) the health state to be summarized; | | ✓ | |
|  |  |  | (c) the measure of occurrence; and | | ✓ | |
|  |  |  | (d) any stratification variables, if applicable. | | NA | |
| Methods | |  |  | |  | |
| Study design | | 5 | (a) State whether the study is cross-sectional or longitudinal. | | ✓ | |
|  |  |  | (b) Restate the measure of occurrence being targeted. | | ✓ | |
|  |  |  | (c) If the study is longitudinal, specify the time origin and follow-up period for the measure of occurrence; if the study is cross-sectional, specify the time anchor at which the health state is summarized for individuals. | | ✓ | |
| Setting | | 6 | Describe any relevant features of the place and time in which the target population resides and across which data were collected. | | ✓ | |
| Participants | | 7 | (a) Describe the target population thoroughly in terms of person, place, and time. | | ✓ | |
|  |  |  | (b) Describe sampling into the study population (whether sampling was explicit or implicit, e.g., by inclusion in an administrative database); this includes eligibility criteria (see recommendations on data sources in item 10 below). | | ✓ | |
|  |  |  | (c) Describe any restrictions on the analytical sample. | | NA | |
| Outcome(s) | | 8 | (a) State when and how the outcome is measured. | | ✓ | |
|  |  |  | (b) Include estimates or discussion of the sensitivity and specificity of the study outcome definition relative to the gold standard. | | NA | |
|  |  |  | (c) List secondary outcomes or competing events of interest. | | NA | |
| Covariates | | 9 | Specify any stratification or adjustment variables—clearly define how variables were collected or constructed. | | ✓ | |
| Data sources/measurement | | 10 | Clearly delineate any inclusion/exclusion criteria for membership in the data source, including the original purpose for which the data were collected, if not for the study at hand. | | ✓ | |
| Bias | | 11 | Describe any assumptions or methods used to extrapolate data from the analytical sample to the study population and from the study population to the target population. | | NA | |
| Statistical methods | | 12 | (a) Describe the primary statistical methods used to estimate the measure of disease occurrence being targeted; discuss assumptions of that method in light of data limitations (e.g., assumption of independent censoring for people lost to follow-up). | | ✓ | |
|  |  |  | (b) If any adjustment/standardization will be done, state the goal of such adjustment. | | ✓ | |
| Results | |  |  | |  | |
| Participants | | 13 | Report numbers of individuals at each study stage (this is likely to be approximate for the target population); consider summarizing this information in a flow diagram. | | ✓ | |
| Descriptive data | | 14 | (a) Report on the characteristics of the analytical sample in a “Table 1.” | | ✓ | |
|  |  |  | (b) Indicate the number of participants with missing data for each variable used in the analysis. | | ✓ | |
|  |  |  | (c) If any weighting or imputation is done to reconstruct the study sample or target populations, include columns for those populations. | | NA | |
| Outcome data | | 15 | (a) Present an overall (unstratified) estimate of the measure of occurrence of interest. | | ✓ | |
|  |  |  | (b) Report “crude” (raw data in the analytical sample) and (if applicable) “corrected” (after any weighting or imputation) estimates. | | NA | |
| Other analyses | | 16 | Present prespecified stratum-specific or adjusted/standardized results. | | ✓ | |
| Discussion | |  |  | |  | |
| Key results | | 17 | Summarize key results with reference to the study objectives. | | ✓ | |
| Limitations | | 18 | Summarize potential sources of selection bias and measurement error and any attempts to mitigate these biases. Discuss both the direction and magnitude of any potential bias. Integrating quantitative bias analysis into the study to guide these discussions is encouraged. | | ✓ | |
| Interpretation | 19 | | | (a) Avoid causal interpretations of descriptive results; avoid overinterpreting stratum-specific differences in measures of occurrence. | | ✓ |
|  |  |  |  | (b) Describe how results of this study might inform or improve public health or clinical practice. | | ✓ |
